# Supplementary material for: Causal Relationship between Aging and Anorexia Nervosa: A White-Matter-Microstructure-Mediated Mendelian Randomization Analysis
Source: Biomedicines. 2024 Aug 16;12(8):1874. doi: 10.3390/biomedicines12081874 (PMC11351342; doi:10.3390/biomedicines12081874)
Supplement: Supplementary file 1 [file biomedicines-12-01874-s001.zip › Supplementary Figure/Figure S4/Singe SNP plot of MR analysis for TL to ubm-b-1713.pdf]

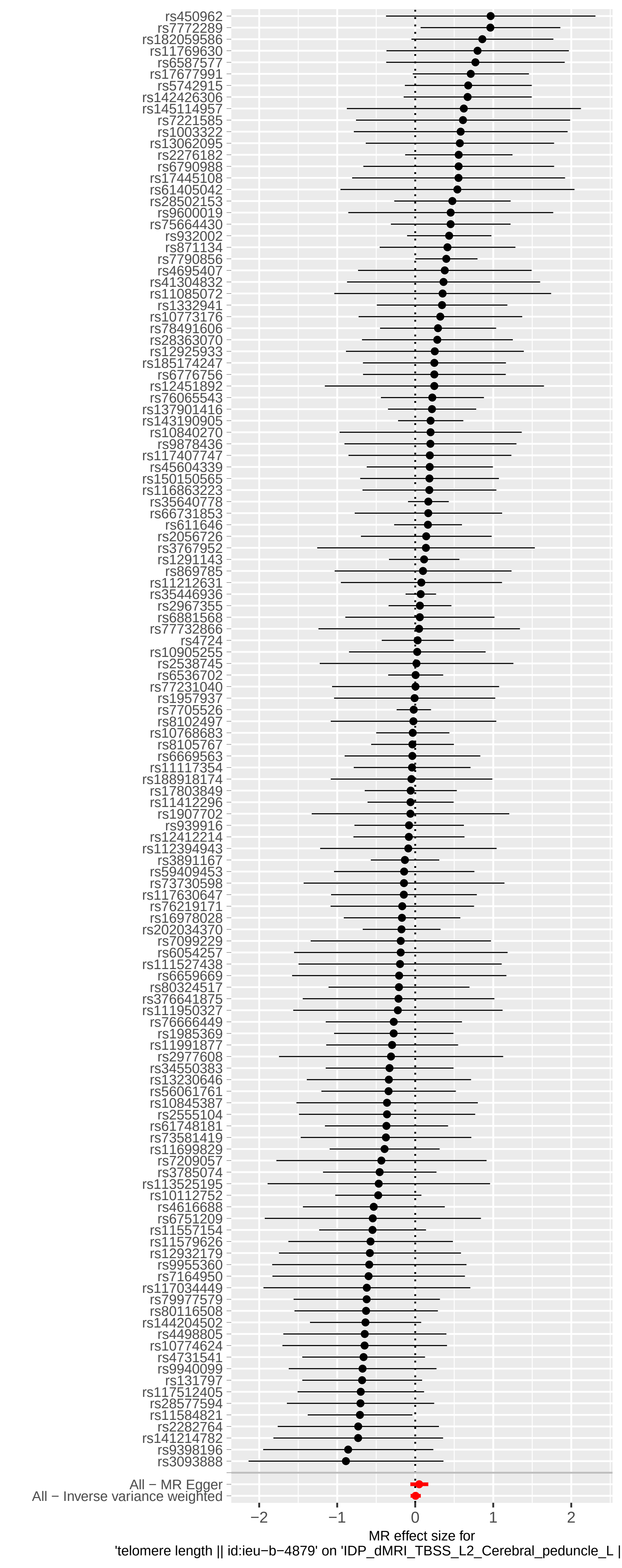
rs450962
rs7772289
rs182059586
rs11769630
rs6587577
rs17677991
rs5742915
rs142426306
rs145114957
rs7221585
rs1003322
rs13062095
rs2276182
rs6790988
rs17445108
rs61405042
rs28502153
rs9600019
rs75664430
rs932002
rs871134
rs7790856
rs4695407
rs41304832
rs11085072
rs1332941
rs10773176
rs78491606
rs28363070
rs12925933
rs185174247
rs6776756
rs12451892
rs76065543
rs137901416
rs143190905
rs10840270
rs9878436
rs117407747
rs45604339
rs150150565
rs116863223
rs35640778
rs66731853
rs611646
rs2056726
rs3767952
rs1291143
rs869785
rs11212631
rs35446936
rs2967355
rs6881568
rs77732866
rs4724
rs10905255
rs2538745
rs6536702
rs77231040
rs1957937
rs7705526
rs8102497
rs10768683
rs8105767
rs6669563
rs11117354
rs188918174
rs17803849
rs11412296
rs1907702
rs939916
rs12412214
rs112394943
rs3891167
rs59409453
rs73730598
rs117630647
rs76219171
rs16978028
rs202034370
rs7099229
rs6054257
rs111527438
rs6659669
rs80324517
rs376641875
rs111950327
rs76666449
rs1985369
rs11991877
rs2977608
rs34550383
rs13230646
rs56061761
rs10845387
rs2555104
rs61748181
rs73581419
rs11699829
rs7209057
rs3785074
rs113525195
rs10112752
rs4616688
rs6751209
rs11557154
rs11579626
rs12932179
rs9955360
rs7164950
rs117034449
rs79977579
rs80116508
rs144204502
rs4498805
rs10774624
rs4731541
rs9940099
rs131797
rs117512405
rs28577594
rs11584821
rs2282764
rs141214782
rs9398196
rs3093888
All − MR Egger
All − Inverse variance weighted
−2
−1
0
1
2
MR effect size for
'telomere length || id:ieu−b−4879' on 'IDP_dMRI_TBSS_L2_Cerebral_peduncle_L |
